# The no-SCAR (Scarless Cas9 Assisted Recombineering) system for genome editing in *Escherichia coli*

**DOI:** 10.1038/srep15096

**Published:** 2015-10-14

**Authors:** Chris R. Reisch, Kristala L. J. Prather

**Affiliations:** 1 Department of Chemical Engineering, Massachusetts Institute of Technology, Cambridge MA 02139.; 2 Synthetic Biology Engineering Research Center (SynBERC), Massachusetts Institute of Technology, Cambridge MA 02139.

## Abstract

Genome engineering methods in *E. coli* allow for easy to perform manipulations of the chromosome *in vivo* with the assistance of the λ-Red recombinase system. These methods generally rely on the insertion of an antibiotic resistance cassette followed by removal of the same cassette, resulting in a two-step procedure for genomic manipulations. Here we describe a method and plasmid system that can edit the genome of *E. coli* without chromosomal markers. This system, known as Scarless Cas9 Assisted Recombineering (no-SCAR), uses λ-Red to facilitate genomic integration of donor DNA and double stranded DNA cleavage by Cas9 to counterselect against wild-type cells. We show that point mutations, gene deletions, and short sequence insertions were efficiently performed in several genomic loci in a single-step with regards to the chromosome and did not leave behind scar sites. The single-guide RNA encoding plasmid can be easily cured due to its temperature sensitive origin of replication, allowing for iterative chromosomal manipulations of the same strain, as is often required in metabolic engineering. In addition, we demonstrate the ability to efficiently cure the second plasmid in the system by targeting with Cas9, leaving the cells plasmid-free.

Genetic engineering through homologous recombination, known as recombineering, has provided new ways to manipulate DNA *in vivo*. In *E. coli*, λ-Red prophage assisted recombineering has facilitated new and easy methods for defined insertions, deletions, and point-mutations^1^,^2^,^3^. Perhaps the most popular method of recombineering described to date is that of Datsenko and Wanner for one-step gene inactivation^1^. In this method an antibiotic resistance cassette is inserted into the chromosome in place of the targeted gene. The target is determined by homology arms that are built into the PCR primers used for amplifying the antibiotic resistance marker and can be as short as 35 bp. This linear dsDNA is then introduced into cells that have the λ-Red genes *bet, exo*, and *gam* expressed to facilitate genome integration^4^. Recombinant cells are selected by antibiotic resistance and location of the marker confirmed by PCR. The antibiotic marker is flanked by flippase recombination targets (FRT) which allows removal of the marker, but leaves a single FRT scar site on the chromosome.

Methods for creating scarless chromosomal point mutations, gene insertions, or promoter replacements also proceed by initially inserting a dual-selectable marker^2^,^5^,^6^,^7^. After insertion of the dual-selectable marker, a second transformation is performed to remove the marker and insert the desired point mutation or gene. Since the targeted gene is initially removed from the chromosome, genes that are essential for cell survival must be expressed in-trans during the process, adding an additional layer of complexity. Alternatively, homing-endonuclease recognition sequences can be inserted into the chromosome as a counter-selectable marker^8^,^9^,^10^. A helper plasmid may then donate DNA through homologous recombination upon double strand break (DSB). After recombination the homing endonuclease recognition sequence is removed, while cells that do not undergo successful insertion are selected against by DSB. Again, essential genes must be expressed in-trans, though it was recently shown that clever designs allow this system to function on essential genes without in-trans expression^10^. While these methods have been used successfully, they suffer from drawbacks that include complicated cloning schemes to add DNA homology, scar sites that destabilize the chromosome, the requirement for multiple rounds of selection for a single manipulation, and low success rates.

Multiplex automated genome engineering (MAGE) was described several years ago as a tool for genome-wide manipulations using oligonucleotide mediated recombination^11^. This method utilizes short ssDNA fragments purchased in the form of oligonucleotides that were shown to recombine with high efficiency with assistance from the λ-red protein Bet^12^. Development of an automated system to continuously cycle a population of cells allowed for rapid changes on the genome scale. The power of this method was demonstrated by removing a single codon from the entire *E. coli* genome^13^. However, MAGE requires an automated system that is commercially unavailable. More recently co-selection MAGE was described as a means to achieve high-throughput genome engineering without the need for the automated system originally described^14^. For co-selection MAGE, several oligonucleotides are simultaneously transformed, including one which confers a selectable phenotype. Cells that possessed the selectable mutation were far more likely to have additional allelic replacements (AR) in targets within close proximity^15^. Utility of this system is limited by the availability of co-selectable markers at regions throughout the genome. A strain could be created which possesses such selectable markers located throughout the genome by recombineering with dsDNA. While possible, strain construction would be difficult and would prevent the use of project specific strains of *E. coli*.

The CRISPR/Cas9 system has been a transformational tool for genome engineering in a variety of organisms^16^,^17^. Cas9 is an endonuclease that targets a specific DNA sequence that can be easily programmed by a plasmid encoded target sequence of 20 bp^18^. The only requirement of the *Streptococcus pyogenes* Cas9 is that the protospacer be adjacent to the triplet NGG, known as the protospacer adjacent motif (PAM). There are 424,651 GG doublets on both strands of the *E. coli* chromosome^19^, making PAM site availability unlikely to limit Cas9 targeting. Cas9 cleavage of genomic DNA results in cell death because *E. coli* lacks the classical non-homologous end joining mechanism for DNA repair^20^. It was shown that the CRISPR Cas9 system can be used to successfully select for cells with point mutations that alter the guide RNA target sequence in both *Streptococcus pneumoniae* and *E. coli*^21^. In *S. pneumoniae*, a naturally recombinogenic strain, this system could be used to make deletions, insertions, and point mutations. In *E. coli*, expression of the λ-Red gene *bet* was required to introduce a mutation into *rpsL*. With Cas9 counterselection this mutation could be selected with an efficiency of 60% of surviving cells. Much more recently, this same two-plasmid system was combined with the λ-Red recombineering plasmid pKD46 and the ability to insert or delete genes from both ssDNA and dsDNA donors at a single chromosomal locus was demonstrated^22^. Others have shown that the *E. coli* genome can be edited using plasmid DNA as a donor with Cas9 counterselection^23^. We sought to extend utility of the Cas9 counterselection method and develop an easy to use plasmid system that could efficiently make point mutations, gene deletions, and short sequence insertions iteratively and with high efficiency.

Here we describe the no-SCAR (Scarless Cas9 Assisted Recombineering) method for genome modifications in *E. coli.* This two-plasmid system contains all of the components required so that host specific modification are not needed. We demonstrate that this method enables the selection of gene deletions, point mutations, and short insertions in a single-step at many genomic locations. No-SCAR, outlined in Fig. 1, can increase the throughput and ease with which complex genome engineering projects are completed. In contrast to other recently described Cas9 counterselection methods we provide easy to perform instructions for sgRNA plasmid retargeting and demonstrate that both plasmids can be easily cured in a few days. Compared to other methods for scarless genome editing that use dual selection cassettes the no-SCAR system takes a similar amount of time and effort when only a single mutation is performed. However, for projects that require more than one mutation the system becomes much faster with subsequent mutations made in as little as three days.

## Methods

### Plasmid construction and protospacer targeting

All plasmids listed in Table S1 were created using circular polymerase extension cloning (CPEC)^24^. The primers listed in Table S2 were used to create linear dsDNA products that were subsequently DpnI digested for at least 15 minutes and then gel-purified. The DNA was eluted in 6 ul of dH_2_O and mixed with corresponding linear product. These products were then used as template for a reaction with Q5 polymerase for 15 cycles. The product was then used to directly transform chemically competent *E. coli* DH5α or NEB Turbo cells. The following plasmids and maps have been deposited to Addgene (Cambridge, MA); pCas9cr4 (Plasmid #62655), pKDsg-ack (Plasmid #62654), and pKDsg-p15 (Plasmid #62656).

**Figure 2:**
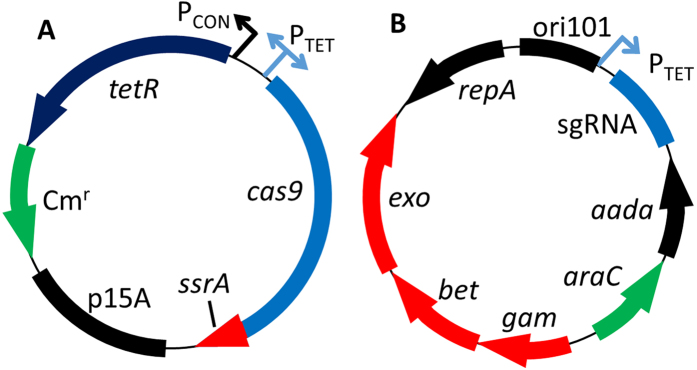
Schematic map of the no-SCAR plasmids. (**A**) Schematic of the plasmid pCas9cr4 which has *cas9* expressed under control of the P_TET_ promoter and *tetR* constitutively expressed. (**B**) Schematic of the plasmid pKDsg-xxx which has the sgRNA expressed under control of the P_TET_ promoter and the three genes that compose the λ-Red system under control of the arabinose inducible promoter P_araB_.

The 20 bp targeting sequences of the sgRNA were re-targeted by CPEC cloning of two linear PCR fragments. The re-targeting primers listed in Table S2 were approximately 40-mers that had overlapping protospacer sequences. The primer pair protospacerF and gamR were used to yield a 3 kb product. The protospacer R primer was paired with pKDseq1F to yield a product of about 4 kb. This design yielded PCR product with about 280 bp of overlapping homology between the *gam* and *araC* (Fig. 2), as well as 20 bp of overlap in the protospacer. PCR products were gel purified, mixed together in equal volumes and CPEC cloned with Q5 polymerase. The mixture was used to transform chemically competent DH5α or NEB Turbo cells (New England Biolabs), recovered for 1 hour in super optimal broth with catabolite repression (SOC), and then plated on LB with 50 mg L^−1^ spectinomycin (spec) and incubated at 30 °C. A step-by-step protocol for primer design and retargeting is available as part of the no-SCAR protocol at Addgene.

### Recombineering

Oligonucleotides used for recombineering should be designed using previously described parameters that maximize efficiency^25^. In our experience the most important factors for successful oligonucleotide recombineering are: proper length of oligo (60–90 bases), 2–4 phosphorothioate bonds to the 5′ end, targeting the lagging strand, and avoiding mismatch repair. The dsDNA recombineering presented in this work did not possess any special modifications. However, efficiency may be increased by adding a phosphate to the 5′ end of the strand that corresponds to the leading strand and/or adding 2–4 phosphorothioate bonds to the 5′ end of the strand corresponding to the lagging strand^26^,^27^.

*E. coli* MG1655 that possessed both the pCas9cr4 plasmid and pKDsgRNA plasmids were grown in 4 mL of super optimal broth (SOB) with 50 mg L^−1^ spec and 34 mg L^−1^ chloramphenicol (cm) at 30 °C. At an OD of approximately 0.5, λ-red was induced with the addition of 50 mM L-arabinose and incubated for 20 minutes. Cells were then made electrocompetent by the glycerol/mannitol density step gradient as described previously^28^. Cells were re-suspended in the glycerol/mannitol solution at 50 μl per one mL of culture. Oligonucleotides were added to 50 μl of cells at a final concentration of 10 μM and then transferred to a 0.1 mm electroporation cuvette. Electroporation was performed on a BioRad Gene Pulser and cells were immediately recovered in 1 mL final volume of SOC for 1–2 hours before plating on LB with 34 mg L^−1^ Cm, 50 mg L^−1^ Spec, and 100 ng mL^−1^ anhydrotetracycline (aTc). Plates were incubated at 30 °C overnight.

The *ack* mutation and deletion was screened by growth on M9 minimal medium plates with 2% glycerol, 10 mM chloroaceate, and 0.1% SOC. Colonies were patched from the overnight plates and onto the selective plates using a toothpick and incubated at 30 °C for two nights. The *rpoB* mutation was screened by patching colonies onto LB with 20 mg L^−1^ rifampicin. Plates were incubated at 37 °C overnight and scored for growth.

The dsDNA for insertion of the *ssrA* tag onto *pfkA* was PCR amplified with primers pfkA Down F and R using strain IB1643^29^ as template.

### Colony counts

Colony forming units were determined similarly to the miniaturized plating method described previously^30^. Briefly, 10 μL of recovered cultures, as well as dilutions of 10^−1^, 10^−2^, and 10^−3^, were spotted on plates in triplicate and incubated overnight at 30 °C.

### Genotyping PCR

Transformants were genotyped by allele specific PCR^31^. Briefly, the 3′ end of the mutant genotyping primer listed in Table S1 annealed perfectly to the mutant genotype, while wild-type possessed mismatched DNA at the 3′ end. Hotstart Taq polymerase that did not possess 3′ to 5′ exonuclease activity was used for colony PCR of the putative mutants. Only those cells which had incorporated the mutation produced PCR product, since the mismatch between the 3′ end of the primer and wild-type genome did not allow for primer extension. The *ack* deletion was screened by using PCR primers up and downstream of the deletion. The *pfkA* ssrA tag was genotyped with the pfkA Down F and R.

## Results

### Inducible cell death by DNA double strand break

Coexpression of *cas9* and single-guide RNA (sgRNA) that targets the *E. coli* chromosome is lethal due to the introduction of chromosomal DSB^21^,^32^,^33^. Even low levels of *cas9* expression are likely to cause cell death and a plasmid-based system which is capable of maintaining both *cas9* nuclease and genome targeting sgRNA has not been described. We hypothesized that low frequency recombineering events, such as sequence deletions and insertions, would benefit from the ability to co-maintain two plasmids. Methods described previously were successful with co-transformation of plasmid DNA and ssDNA, though this method is inherently less efficient because the cells would need to acquire both the ssDNA and plasmid^21^,^34^. Thus, we set out to design a system with tight control of *cas9* and sgRNA expression to allow maintenance of both plasmids. Induction of *cas9* and sgRNA expression will then cause cell death.

The previously described nuclease-null plasmid pdCas9-bacteria that possessed the *S. pyogenes cas9* was first modified to re-activate nuclease activity creating plasmid pCas9^35^. The pCas9 plasmid possessed the aTc inducible promoter P_TET_ to drive expression of both *tetR* and *cas9.* In the absence of inducer, TetR represses transcription of both *cas9* and *tetR*. However, even in the absence of aTc, co-transformation of pCas9 and sgRNA targeting the *ack* gene in *E. coli* had very low transformation efficiency, while transformation with an off-target sgRNA with pCas9 had an efficiency over two orders of magnitude higher. This suggested that in the absence of inducer leaky expression of *cas9* and sgRNA caused cell death.

To maintain tighter control of *cas9* expression an *ssr*A consensus tag was added to the C-terminus of c*as9*, creating plasmid pCas9cr1. The SsrA tag is recognized by ClpP protease and should speed degradation of Cas9 that results from leaky transcription. Next, plasmid pCas9cr2 was created by adding a constitutive promoter upstream of *tetR* to increase expression of the negative-regulator and maintain tighter transcriptional control. Lastly, the ribosome binding site (RBS) was modified to decrease the theoretical rate of translation initiation by tenfold^36^, creating plasmid pCas9cr4. The plasmid pCas9cr4 was still unable to co-transform with the sgRNA plasmid. However, when cells were first transformed with the pCas9cr4 plasmid and subsequently transformed with the sgRNA encoding plasmid, colonies were obtained in the absence of inducer. The sequential transformation allowed sufficient amounts of TetR to accumulate and effectively repress *cas9* expression.

The sgRNA cassette targeting *ack* was moved from the previously described pgRNA-bacteria plasmid^35^ into the pKD46 plasmid that possessed the λ-Red system and the temperature sensitive origin of replication from pSC101 (Fig. 2)^1^. Initially the sgRNA cassette was placed downstream of the λ-Red genes and upstream of *repA*. However, initial experiments showed that this location was susceptible to recombination events that removed the sgRNA and caused counter-selection escape. Consequently, the sgRNA cassette was placed between the origin of replication and antibiotic marker, to limit the possibility of recombination since both of these components were essential to the plasmid. The ampicillin resistance gene *bla* was also replaced with the spec resistance gene *aadA* to avoid satellite colony formation associated with ampicillin resistance, creating plasmid pKDsg-ack. To further control the inducible cell death phenotype the P_TET_ promoter was inserted in-place of the constitutive promoter that was driving expression of the sgRNA. In the absence of inducer these modifications enabled cell growth that was similar to the plasmid-free parent strain.

While most cells were killed by expression of *cas9* and genome targeting sgRNA, some cells were able to escape the counterselection. To determine the rate of counterselection escape in cells that possessed both plasmids the number of CFU’s after plating was determined in the presence and absence of inducer. As summarized in Table 1, the rate of escape varied from 8.5 × 10^−5^ to 7.2 × 10^−4^ with four different guide RNA targets, with an average of 2.6 × 10^−4^. Even at the high end of the escape frequency, standard point mutations and deletions should be easily identified given the reported frequency of these allelic replacements^11^,^12^,^37^. This escape rate of 2.5 × 10^−4^ was similar to the rate previously determined in *E. coli* and *Lactobacillus lactis*^21^,^34^. In Jiang *et al.* the plasmids were constructed with the native *S. pyogenes* dual guide RNA, in which the plasmid encoded guide sequence was flanked by short palindromic repeats. In this case the escape mutants were found to have lost the guide sequence, likely the result of recombination events in the palindromic repeats. In the system presented here, single-guided RNA was used, which removed these palindromic repeats^18^. In all escape plasmids that were examined, the guide sequence remained intact. The chromosomal protospacer also remained unmodified, making the genetic basis for these escapes unclear. It is possible that point mutations in *cas9* or elsewhere in the sgRNA plasmid alter the level of protein and transcript in the cell, resulting in the escape phenotype. In the cases that we’ve tested this escape phenotype is maintained when plasmids are purified from an escapee and used to transform wild-type MG1655. Nevertheless, this escape rate was low enough that our system could be successfully used to enrich for mutant populations as described below.

### Point mutation selections

**Figure 3:**
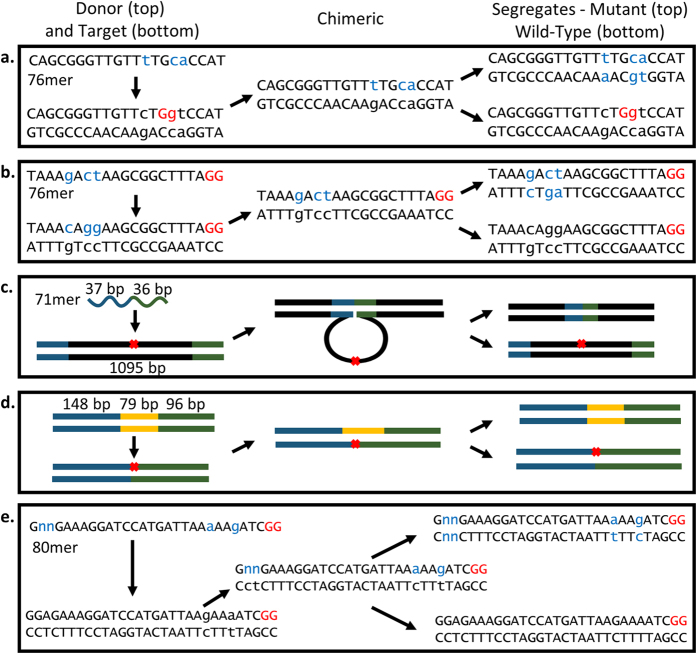
Schematic of the genomic DNA modifications demonstrated by the no-SCAR system. The donor and target DNA are on the left, the chimeric genomic DNA produced after integration through a fully single-stranded DNA intermediate is in the middle, and the segregated chimeric DNA that results in populations of mutant and wild-type are on the right. (**a**,**b**) 21 bp of the targeted region for making point mutations into the *rpoB* and *ack* genes. The PAM site is shown in red, mutations are shown in lowercased blue text, and mutation target in lowercased black text. (**c**) Deletion of 1095 bp of *ack* using a 73mer oligo. (**d**) Insertion of 79 bp at the C-terminus of *pfkA* using dsDNA. Red × indicates the site of Cas9 targeting. (**e**) Insertion of degeneracies outside of the protospacer sequence.

The system was first tested for the ability to counterselect against wild-type cells when inserting point mutations during oligonucleotide mediated recombineering. Oligonucleotides were designed to insert point mutations into the *ack* and *rpoB* genes of *E. coli* (Fig. 3a,b). The point mutations were designed to alter the PAM sequence or protospacer sequences within 12 bp of the PAM because this region was shown to be most important for Cas9 specificity^38^. The oligonucleotides were targeted to the lagging strand, since the efficiency of allelic replacement is over two orders of magnitude greater than the leading strand^12^. The *ack* mutation was designed to insert a nonsense mutation which gives resistance to the acetate analog chloroacetate. The *rpoB* mutation resulted in a missense point mutation, D516V, which confers resistance to rifampicin^39^.

For the *ack* mutation, the system was tested with two different transformation schemes. First, cells which possessed both the pKDsgRNA-ack and *cas9* encoding plasmids were transformed with oligonucleotide. Alternatively, cells that possessed only the pKDsgRNA-ack encoding plasmid, which also carries the λ-Red genes, were co-transformed with pCas9cr4 and oligonucleotide. In both cases, cells were grown to mid-logarithmic phase, λ-Red was expressed for 20 minutes, and then electrocompetent cells were prepared. Cells were then transformed with oligonucleotides, recovered for 1–2 hours, and then spread onto plates with aTc to express *cas9* and sgRNA. Controls were performed in which cells were transformed with oligonucleotides that did not match the sgRNA guide that it possessed. For the cells that possessed both plasmids before transformation with the oligonucleotides there were 60-fold (3.91 × 10^5^/6.67 × 10^3^) more CFU’s mL^−1^ with ackmut2 than the control transformation (Table 2), suggesting efficient selection for only those cells that incorporated the desired mutation. Putative *ack* mutants and controls were patched onto M9 plates with 10 mM chloroacetate and glycerol as the sole carbon source. After a 48 hour incubation the results showed that 99 ± 1% of the putative point mutants grew on the selective plates while only 8.1 ± 0.9% of the control transformants grew. The colonies from the control plate that grew were escape mutants that became spontaneously resistant to chloroacetate. Thus, up to 8.1% of the point mutants could actually be escapes and not possess the desired mutation. However, allele specific PCR of 24 randomly selected chloroacetate resistant colonies showed that all 24 possessed the desired mutation (Figure S1), consistent with the colony counts presented in Table 2 which predicted that only one in 60 colonies were wild-type for *ack*.

The alternate protocol, in which both pCas9cr4 and the oligonucleotide were co-transformed, yielded a 4.6 fold change in CFU’s (2.3 × 10^2^ / 0.5 × 10^2^), though significantly fewer colonies were obtained. Each colony was genotyped using allele specific PCR. The results showed that 21 of 24 colonies possessed the desired mutation. Thus, using this double transformation protocol it was possible to select for point mutations with high efficiency.

The *rpoB* mutation was tested by the single transformation protocol only. Cells that possessed both pCas9cr4 and pKDsgRNA-rpoB were transformed with the rpoBmut or ackmut2 and plated on LB with aTc. There were 15-fold more colonies with the on-target oligonucleotide than the off-target (1.7 × 10^5^/1.1 × 10^4^). Colonies from both the experimental and control transformations were patched onto LB plates with rifampicin. After 1 day of incubation 94 ± 4% of the putative mutants grew while only 3 ± 1% of the control transformants grew. The number of positive colonies from the rifampicin plate was consistent with the colony counts which predicted that 1 of 16 (93.75%) colonies would be Cas9 escape mutants. To find the total mutation frequency, a similar experiment was performed in which the recovered cells were plated on medium with and without inducer. The total mutation frequency was found to be 2.3 ± 1.3% of cells that survived electroporation. This efficiency was slightly lower than the efficiencies reported elsewhere^11^,^40^, but still about two orders of magnitude above our escape rate.

In addition to the mutations described above, point mutations have been made in the genes *apt, gyrA, ppc, pck, rpsL, pts*, and *yjgB*. The success and efficiency of creating these point mutations suggested that the system is robust enough to make point mutations in any genomic location with an appropriate PAM site.

### Oligonucleotide mediated deletions

Next, the system was tested for its ability to select for deletions of chromosomal DNA using oligonucleotides with upstream and downstream homology to the area of deletion. It was previously shown that up to 45 kbp could be deleted from the chromosome by a single 90mer oligonucleotide, though the frequency of recombination decreased as the size of deletion was increased^11^. We used the same *ack* targeting sgRNA as described in the previous section and a 71 bp oligonucleotide that was designed to delete 1095 bp of the *ack* gene (Fig. 3c), thereby removing the targeted protospacer. Again, both the single and double transformation protocols were tested. The single transformation protocol, in which the cells possessed both *cas9* and sgRNA-ack encoding plasmids, yielded approximately 8-fold more CFU’s (5.5 × 10^4^/6.6 × 10^3^) when transformed with on-target oligonucleotides than off-target. Colonies were patched from the original plate and onto the chloroacetate selection plate where 85.6 ± 6.4% of colonies grew. Colony PCR of 12 randomly selected colonies showed that all 12 possessed the deletion (Figure S2). For the double transformation protocol there were 2.4-fold more colonies with the on-target oligonucleotide than the control (2.2 × 10^2^ / 0.5 × 10^2^). Colony PCR of the putative mutants showed that 60% had the deletion.

In addition to the deletion of *ack*, this method was used to successfully delete 500 bp of the *sspB* gene and 3 kbp spanning the *dkgA-yqhD-yqhC* locus. Colony PCR showed that 42% of the *sspB* and 16% of the larger *dkgA-yqhD-yqhC* (Figure S3) colonies possessed the correct deletions. The efficiency of oligonucleotide mediated deletions is known to be inversely related to the length of the deletion^11^. A deletion of 45 kbp was obtained at an efficiency of between 0.01–0.1 percent. Given the escape rate that was determined here, 2.6 × 10^−4^, even very large deletions should be possible with this method.

### Short sequence insertions

Next, the ability to insert short sequence motifs was examined. It was previously demonstrated that oligonucleotides could be used to insert up to 30 bp into the chromosome^11^. The size of insertion using an oligonucleotide is limited due to size constraints of the oligonucleotide itself. As an alternative to ssDNA, dsDNA sequences could also be used to insert short fragments into the chromosome. The no-SCAR system maintains *exo* and *gam* from λ-Red which facilitate chromosomal integration through single stranded intermediates at the replication fork in a mechanism analogous to oligonucleotide recombineering^26^,^27^. Linear dsDNA has recently become available as gBlocks from Integrated DNA Technologies (Coralville, IA) and DNA strings from Life Technologies (Carlsbad, CA) for an economical price with delivery in just a few days. Fragments can be designed *de novo* and the homology required for targeting included in the sequence. This alleviates the need to incorporate homology on PCR primers, thereby allowing longer regions of homology to be used, which can increase the efficiency of insertion^27^. Since the mass of these fragments is typically only 200–500 ng, the fragment should be PCR amplified before transformation. To test the ability of our system to select for dsDNA mediated mutants, we PCR-amplified a 300 bp fragment that also contained a 79 bp *ssrA* tag insertion at the C-terminal of the *pfkA* gene (Fig. 3d). The fragment included 80 bp of upstream and 140 bp of downstream homology to the insertion site. After transformation there were approximately 4-fold more colonies after transformation with the on-target dsDNA (8.05 × 10^−5^/2.1 × 10^−5^). However, PCR genotyping of three independent experiments found that 36 out of 36 colonies possessed the 80 bp insertion (Figure S4). Thus, the efficiency of insertion was higher than the colony counts would predict. The insertions were further confirmed by sequencing of 5 clones, all of which had the predicted insertion (Figure S5). While we did not probe the upper limits of insertion size, it is known that recombination efficiency decreases with increasing insertion size^27^. However, it should be possible to identify insertions of over 1 kb using the no-SCAR system. Insertion of a 1.2 kb fragment with 45 bp homology arms had an insertion efficiency of 1.9 × 10^−4^ recombinants per viable cell^26^. Given our escape rate of 2.6 × 10^−4^, similar insertions should be easily identified after Cas9 counterselection.

### Chromosomal degeneracies

The examples presented above demonstrated the ability to select for mutations that alter or remove sequences within 12 bp of the PAM site. We next sought to create an RBS library by including degeneracies in the oligonucleotide while targeting a PAM site that was outside of the 12 bp window of specificity (Fig. 3e). A PAM site located 13 bp downstream of the start codon was targeted, which allowed synonymous mutations to be inserted within 12 bp of the PAM site and disrupt Cas9 targeting. In addition, two N-degeneracies that were 26 and 27 bp from the PAM site were incorporated into the oligonucleotide used for mutagenesis. After transformation of the degenerate oligonucleotide, 23 putative mutants were sequenced (Figure S6). Of these 23 sequences 11 possessed the silent mutations and eight of these also possessed an altered RBS. There were three possible explanations for the three sequences that possessed the silent mutations but not the RBS alterations; the degeneracies could have simply matched the wild-type, oligo degradation by DNA Polymerase I or III could have removed the degeneracies^41^, or methyl-directed mismatch repair could have caused reversion to wild-type after oligo incorporation. Since mismatch repair is a possibility it may be prudent to design the mutations which fall outside of the protospacer target to also avoid mismatch repair. Though we have not tested the limits of how far away these additional mutations can be made, the possibility of DNA degradation and host mismatch repair will affect these efficiencies. Regardless, this experiment demonstrated that mutations can be made outside of the 15 bp window of the PAM site and adjacent sequence that is most important for Cas9 targeting. Moreover, the ability to insert degeneracies directly onto the chromosome and produce a genome encoded DNA library was demonstrated.

### Plasmid curing

Upon completion of genome engineering projects removal of the no-SCAR plasmids allows for plasmid origins and markers to be used again for various purposes. The pKDsg-xxx plasmid can be cured by growth at 37 °C but pCas9cr4 did not possess an inherent property that allowed curing of the plasmid. We hypothesized that the pCas9cr4 plasmid could be cured by introducing DSB through Cas9 cleavage. To test this hypothesis the pKDsg-p15A plasmid was created which targeted the p15A origin of replication of pCas9cr4. Upon transformation of pKDsg-p15A into cells that contained pCas9cr4 the cells were recovered in SOC for 1 hour, then 100 ng mL^−1^ aTc was added and incubated for an additional 2 hours before plating on LB with Spec and aTc. The resultant colonies were patched onto LB plates with and without chloramphenicol (Cm) and grown at 37 °C. After overnight growth all patched colonies grew on the LB only plate and had no growth on the Cm plate, indicating loss of the pCas9cr4 plasmid. Furthermore, plasmid mini-preps did not yield either plasmid, confirming that both plasmids were cured. This technique could be used to cure other plasmids as well.

### Comparison with other scar-free genome editing techniques

To demonstrate the speed and utility of this method a day-by-day comparison of the no-SCAR method with two other recently published methods for scarless genome modifications and the method of one-step gene inactivation from Datsenko and Wanner was performed (Table 3). The two recently described Cas9 counterselection techniques^21^,^22^ were not included because they do not demonstrate the ability to remove the sgRNA or *cas9* encoding plasmids, making them unsuitable for iterative modifications. The scheme in Table 3 takes into account the time required for cloning and assumes that wild-type cells were used and λ-Red is not integrated into the chromosome. As demonstrated in Table 3, all four systems take a similar number of days for completion of a single modification when starting and ending with cells that are plasmid-free. When more than one modification is required the no-SCAR system was faster than the compared methods since the pCas9cr4 plasmid remained in the cells after the first iteration. For example, if 3 genome modifications were undertaken and all sgRNA cloned simultaneously, the no-SCAR method would take as little as 14 days, while the next fastest method would take as little as 18 days. Time savings continue to accumulate as additional mutations are made. Moreover, the no-SCAR method is the only one in which essential genes can be targeted without additional constraints since the wild-type gene is never removed from the chromosome.

## Discussion

Recombineering in *E. coli* with ssDNA was first described over ten years ago^12^. Based upon the belief that ssDNA tails were important intermediates in dsDNA recombineering, it was hypothesized that ssDNA alone may be capable of chromosomal recombination. Indeed, it was found that 70mer oligonucleotides were capable of introducing point mutations and deletions of 3.3 kb in the chromosome. Oligonucleotides that corresponded to Okazaki fragments, termed lagging strand, had a much higher efficiency for these allelic replacements. In subsequent years several additional modifications were identified that continued to increase the efficiency of ssDNA recombineering. Some of these modifications were manifested in the design of the oligonucleotides, such as increased length, use of phosphorothioate bonds, and limiting the secondary structure^11^. Other modifications were made to the host strain, including inactivation of the mismatch repair system^40^, removal of endogenous nuclease genes^42^, and alteration of replisome dynamics^43^. While these host strain modifications have increased the efficiency of ssDNA recombineering, they come at a cost of increased host-strain mutations and reduced cell fitness. A host-strain with these modifications is available through Addgene; however, using this pre-designed strain prevents the use of project-specific strains.

Disabling the host mismatch repair system was the first modification that was observed to have a significant impact on the efficiency of allelic replacement^40^. A *mutS* deletion strain increased the frequency of allelic replacement by over 100-fold^40^. Consequently, most studies have since relied on strains deficient in mismatch repair. Unfortunately, *mutS* deletion strains accumulate background mutation rates that are 100-fold higher than wild-type *E. coli*^44^. Though most of these background mutations are likely to be innocuous, some may have deleterious effects on cell fitness or desired phenotype. Recently a strain was developed which allowed for temperature sensitive control of host-mismatch repair gene expression^45^. While this strain or other mismatch repair deficient stains may be used with the no-SCAR system, they still suffer from the aforementioned drawbacks. We note that all of the work presented here was performed in strains with the mismatch repair system intact. The *ack* and *rpoB* point mutants were designed to include a C:C mismatch, which results in higher efficiencies due to mismatch repair evasion^40^. When possible, the oligonucleotide should be designed to include a C:C mismatch within six bp of the desired change^37^.

The no-SCAR method described here allows for single-step genome alterations without the use of a selectable marker. This method builds on previous work which demonstrated that Cas9 could counter-select against wild-type *E. coli* and enrich for a point-mutation with an efficiency of 60%^21^. This previous work relied on co-transformation of the guide RNA encoding plasmid along with mutation encoding oligonucleotides. The no-SCAR system was designed to stably maintain plasmids with *cas9* and sgRNA that targets the host-cell chromosome. Several modifications were required to achieve a level of regulation that prevented constitutive cell death. This system used the P_TET_ promoter to control transcription of both the sgRNA and *cas9*. The P_TET_ promoter was repressed by the TetR regulator and it was essential that TetR be present in cells before both sgRNA and *cas9* were introduced into the same cell.

A direct comparison of the plasmids described here with those described previously in Jiang *et al.* was not performed. One key difference between these systems was that the no-SCAR plasmids possess all system components, including λ-Red, and has no host specific requirements. The efficiency of the no-SCAR system was examined using two different transformation protocols. First, a protocol similar to that of Jiang *et al.*, where the host cells possessed one plasmid and were then co-transformed with the second plasmid and mutation conferring oligonucleotide was tested. Second, cells that possessed both no-SCAR system plasmids were transformed with mutation-conferring oligonucleotides only. Both protocols were tested for making point mutations and gene deletions and in both cases a majority of transformants possessed the desired mutations. The efficiency of obtaining point mutations was similar for both protocols. For the less frequent recombination event of making gene deletions, the co-transformation protocol was slightly less efficient. Thus, to save time it is possible to only transform with the λ-Red encoding plasmid and then subsequently co-transform oligonucleotide and pCas9cr4. However, for less efficient modifications we suggest using cells that have both no-SCAR plasmids in order to maximize the likelihood of obtaining correct mutants.

The description of co-selection MAGE provided a framework for medium-throughput genetic manipulations in *E. coli* 15. In the absence of selectable markers available at well-spaced positions around the chromosome, the co-selection MAGE technique would be difficult to use for projects of large scale. The no-SCAR system can be used to make any gene or intergenic region with a PAM sequence a counter-selectable marker. Thus, the no-SCAR method integrates seamlessly with co-selection MAGE. However, we envision this system as a powerful tool to achieve medium to high-throughput genomic modifications in *E. coli* even without co-selection MAGE.

The no-SCAR method can be used for the sequential introduction of a potentially limitless number of modifications. In contrast to the traditional method of λ-Red recombination where the antibiotic marker is removed by Flp recombinase, this method does not leave recombinase recognition site scars, which can cause chromosomal instability and unwanted genomic rearrangements^1^. In contrast to recently described methods that combine Cas9 counter-selection with λ-Red recombineering, our system allows for the simple curing of the sgRNA encoding plasmid by means of its temperature sensitive origin of replication, allowing for transformation of a subsequent sgRNA encoding plasmid. Re-targeting the protospacer can be performed with very high efficiency using ligation independent cloning methods such as CPEC or Gibson assembly^24^,^46^. The first iteration of the no-SCAR method described here requires 5 days, including the cloning step required for sgRNA targeting. Subsequent iterations can be completed in as little as 3 days since the cells already contain the pCas9cr4 plasmid, which is faster than any other method published.

Continued development of this system could allow for simultaneous selection against more than one target. DNA synthesis and ligation independent cloning methods become difficult with highly repetitive sequences required for multiple sgRNA within a single plasmid. Moreover, the frequency of escape due to plasmid recombination and loss of protospacer sequences is also a challenge that must be addressed. In its current state, the no-SCAR system should facilitate genome engineering projects that require several to dozens of modifications.

## Additional Information

**How to cite this article**: Reisch, C. R. and Prather, K. L. J. The no-SCAR (Scarless Cas9 Assisted Recombineering) system for genome editing in *Escherichia coli*. *Sci. Rep.* **5**, 15096; doi: 10.1038/srep15096 (2015).

## Supplementary Material

Supplementary Information

## Figures and Tables

**Figure 1:**
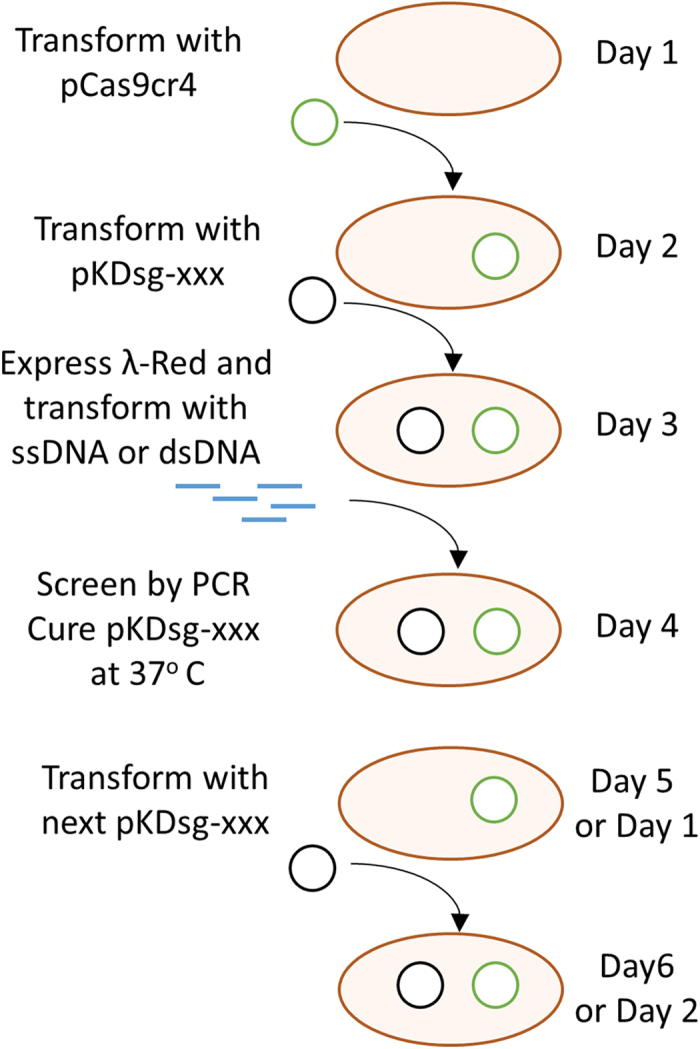
General outline of the no-SCAR method. On day 1 the pCas9cr4 plasmid is used to transform *E. coli*, followed by plating on LB + Cm, and growth at 37 °C. On day 2 the resulting strain can be transformed with pKDsg-xxx plasmid, where –xxx denotes the targeted gene, plated on LB + Spec and Cm, and incubated at 30 °C overnight. On day 3 the resulting strain is grown in SOB until OD ~0.5 and λ-red is induced with 50 mM L-arabinose. After 15–20 minutes the cells are made electrocompetent and transformed with ssDNA or dsDNA that confers a mutation to the protospacer or PAM sequence. After 1–2 hours of recovery the cells are plated on LB + Spec, Cm, and aTc, then incubated at 30 °C overnight. On day 4 colonies are screened by PCR and grown at 37 °C to cure the pKDsg-xxx plasmid. The next pKDsg-xxx plasmid is then used to transform the mutant strain on Day 5 and the process is repeated.

**Table 1:** Fold-change in CFU’s of sgRNA transformants when plated with and without aTc.

Plasmid	Escape Rate^1^
pKDsg-ack	1.16 × 10^−4^
pKDsg-apt	8.49 × 10^−5^
pKDsg-sspB	7.16 × 10^−4^
pKDsg-yqhD	1.13 × 10^−4^

^1^Average of three independent experiments.

**Table 2:** Efficiency of genome editing using the no-SCAR system.

Plasmid	Mutation DNA	CFU’s^1^	Positives^2^	Control DNA	CFU’s^1^	Positives^2^	Fold-Change in CFU’s^3^
pKDsg-ack	ack mut2	3.9 × 10^5^	99 ± 1%	rpoB mut	6.6 × 10^3^	8 ± 1%	61 ± 23
pKDsg-ack	Ack CD	5.5 × 10^4^	85 ± 1%	ack mut2	6.6 × 10^3^	8 ± 1%	9.3 ± 1.9
pKDsg-rpoB	rpoB	1.7 × 10^5^	94 ± 4%	Ackmut2	1.1 × 10^4^	3 ± 1%	16.6 ± 1.8
pKDsg-pfkAE	dsDNA-ssrA	8.0 × 10^5^	100%	dsDNA-off	2.5 × 10^5^	nd	4.5 ± 1.8

^1^The CFU’s are the average of three independent experiments.

^2^The % of positive were obtained from chloroacetate and rifampicin selection plates.

^3^The Fold-Change in CFU’s is the average of the same three independent experiments ± SD.

**Table 3:** Comparison of genome editing techniques.

	No-SCAR	TetA-SacB Dual Selection^5^	SceI counter-selection^10^	Datsenko and Wanner^1^
Day 1	1) Transform pCas9cr4 2) Clone spacer (2 fragments)^1^,^3^	Transform λ-Red plasmid	1) Transform λ-Red plasmid 2) Clone Mutation Cassette (4 fragments)^2^,^3^	1) PCR donor DNA 2) Transform λ-Red plasmid pKD46 into target strain
Day 2	Grow clones	Grow cells overnight	Screen and sequence clones	Transform Linear DNA
Day 3	Isolate plasmid and transform into cells with pCas9cr4	1) Subculture and induce λ-Red 2) Transform TetA-SacB Cassette	PCR amplify Mutation Cassette and Transform	Screen Ab^R^ colonies Cure λ-Red
Day 4	1) Start culture and induce λ-Red 2) Transform linear DNA, induce Cas9	Restreak colonies on counter-selection medium	Restreak Colonies	Transform pCP20
Day 5	1) Screen colonies 2) Grow at 37 to cure pKDsgRNA^4^	Identify sucrose^s^ clones and start overnight culture	Resuspend colonies and plate to induce DSB	Express Flp recombinase
Day 6	Transform pKD-p15, induce Cas9	1) Start Culture and induce λ-Red 2) Transform with linear DNA and plate on counterselection medium	Patch colonies to screen for Ab^S^	PCR screen or Patch for Ab^S^ Grow at 37 to cure plasmid
Day 7	Patch colonies for cm^s^, Grow at 37°	Incubate at 42°	Passage cells to cure plasmid^4^	Plasmid free colonies
Day 8	Plasmid free colonies	Screen by PCR or Tet^S^	Plasmid free colonies	-
1 mutation	5 Days	8 Days	7 Days	6 Days
1 mutation w/ curing	8 Days	8 Days	8 Days	7 Days
2 mutations w/ curing	11^3^ Days	16 Days	13^4^ Days	13 Days
3 mutations w/ curing	14 Days	24 Days	18 Days	19 Days
Essential Genes	No additional requirements	Must express in-trans	Special considerations	N/A

^1^Requires 2 fragment ligation independent cloning, with the primer designs given in the manuscript we have been 100% successful in producing these clones.

^2^Requires 4 fragment ligation independent cloning that should be sequence verified before transformation.

^3^When multiple mutations are desired cloning can be performed simultaneously on day 1, resulting in a faster turnaround time for subsequent mutations.

^4^For more than one mutation the strains obtained during day 5 can be cured of plasmid and immediately transformed with new pKDsgRNA, resulting in 3 day turnaround for each additional mutation.

^5^For more than one mutation the strain obtained on day 7 can be transformed with Linear DNA (Day 3) resulting in a 5 day turnaround for each additional mutation.
