# Supplementary material for: The no-SCAR (Scarless Cas9 Assisted Recombineering) system for genome editing in Escherichia coli
Source: Sci Rep. 2015 Oct 14;5:15096. doi: 10.1038/srep15096 (PMC4604488; doi:10.1038/srep15096)
Supplement: Supplementary Information [file srep15096-s1.pdf]

The no-SCAR (Scarless Cas9 Assisted Recombineering) system for genome editing in *Escherichia coli.*

Chris R. Reisch[1] and Kristala L. J. Prather[1,2,*]

[1]Department of Chemical Engineering, [2]Synthetic Biology Engineering Research Center (SynBERC)

Massachusetts Institute of Technology, Cambridge MA 02139

*To whom correspondence should be addressed: kljp@mit.edu

Supplementary Information

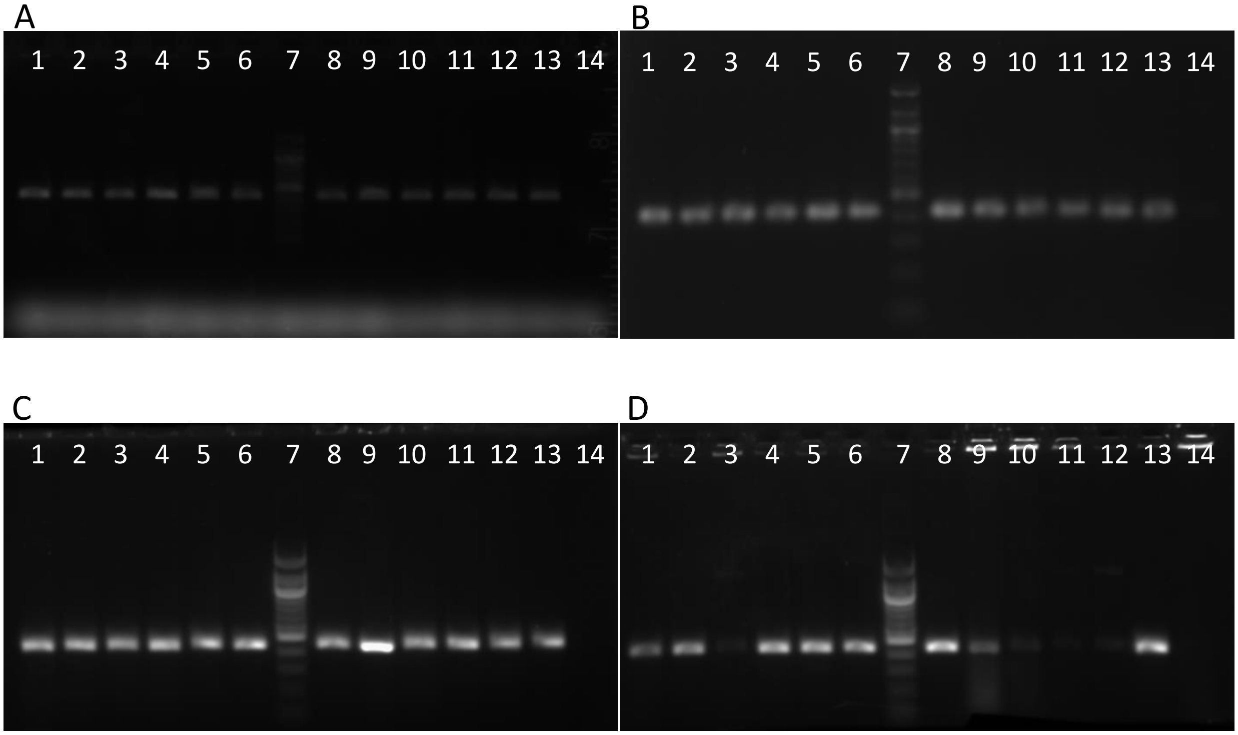

Figure S1. Allele specific genotyping PCR of *ack* point mutations. PCR products were separated on a 1.6% agarose gel. (Top) Colonies from cells that possessed both the pCas9cr4 and pKDsgRNA-ack before transforming ackmut2 oligo. (Bottom) Colonies from cells that possessed only the pKDsgRNA-ack plasmid and was then co-transformed with pCas9cr4 and ackmut2. The colonies from lanes 3, 10, 11, and 12 were re-screened a second time and 3 of the 4 confirmed to be wild-type. Lane 14 was the wild-type control in which no PCR product was observed. (Lane 7) 100 bp DNA ladder (NEB).

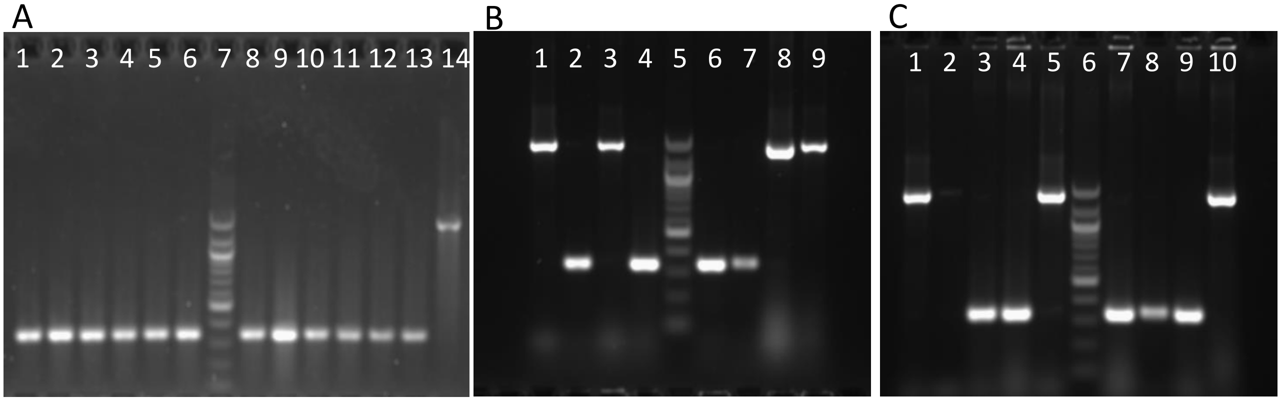

Figure S2. PCR detection of *ack* deletion mutants. PCR products were separated on a 1.6% agarose gel with a 100 bp DNA ladder (NEB). (A) Colonies from cells that possessed both the pCas9cr4 and pKDsgRNA-ack before transforming ackCD oligonucleotide. Lane 14 possessed a wild-type control. (B + C) Colonies from cells that possessed only the pKDsgRNA-ack plasmid and were then co-transformed with pCas9cr4 and ackCD.

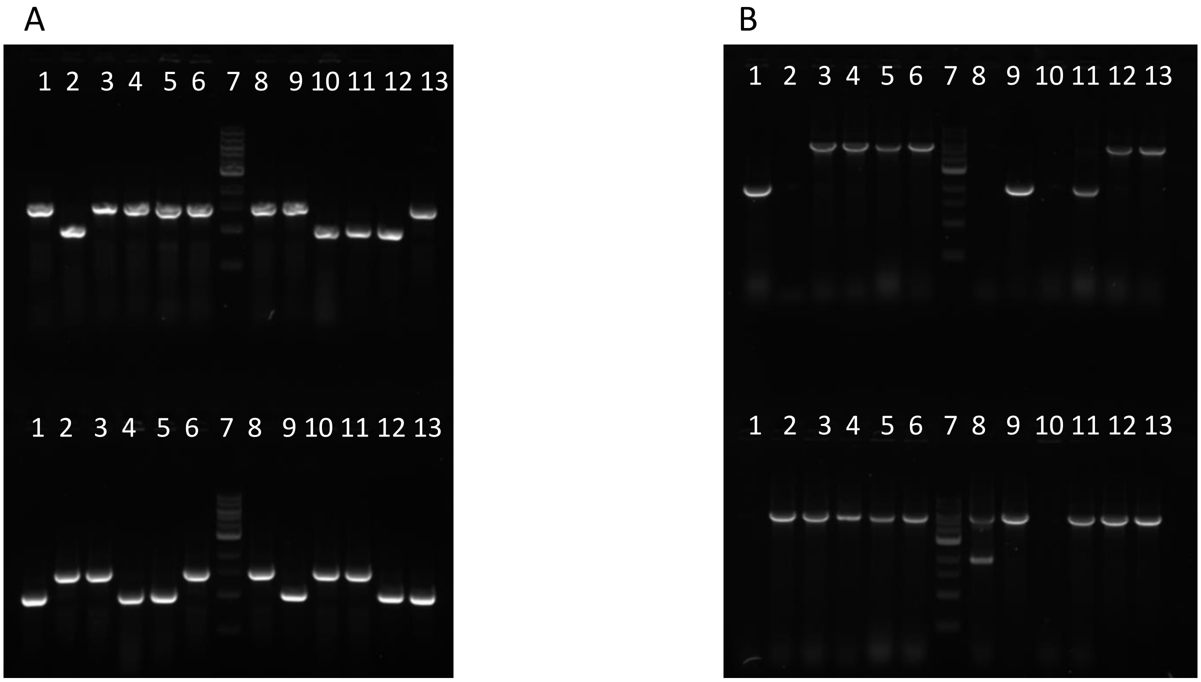

Figure S3. PCR detection of genomic *sspB* and *yqhC-yqhD-dkgA* deletions. PCR products were run on a 0.8% agarose gel. (A) Twenty-four colonies were screened to detect the 477 bp deletion of *sspB*. Ten colonies possessed PCR products of 900 bp, indicating successful deletion of *sspB,* and the remaining 14 colonies had wild-type size of 1400 bp. (B) Twenty-four colonies were screened for the 3200 bp deletion of *yqhC-yqhD-dkgA.* Fifteen colonies possessed wild-type PCR product of 5 kbp, three possessed the 2.2 kpb deletion with a 2 kbp product, one had mix of both products indicating incomplete segregation of the chimeric genotypes, and the PCR reaction failed in 5 of the reactions. A one kb ladder is in lane 7 of each gel.

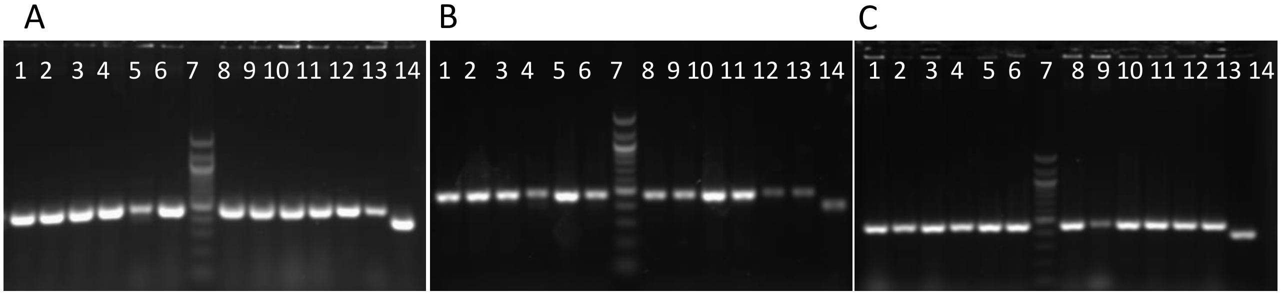

Figure S4. PCR detection of genomic ssrA tag insertion at *pfkA*. PCR products were separated on a 1.6% agarose gel with 100 bp ladder in lane 7. (A,B,C) Twelve colonies from three independent experiments were screened by PCR sizing. The wild-type control in lane 14 in each gel shows a size of 391 bp, while all of the experimental colonies have a size increased by 80 bp.

```
1 TCGGTTTCAGGGTAAAGGAATCTGCCTTTTTCCGAAATCATT-------- 42
2 TCGGTTTCAGGGTAAAGGAATCTGCCTTTTTCCGAAATCATACGGCCCCA 50
3 TCGGTTTCAGGGTAAAGGAATCTGCCTTTTTCCGAAATCATACGGCCCCA 50
4 TCGGTTTCAGGGTAAAGGAATCTGCCTTTTTCCGAAATCATACGGCCCCA 50
5 TCGGTTTCAGGGTAAAGGAATCTGCCTTTTTCCGAAATCATACGGCCCCA 50
6 TCGGTTTCAGGGTAAAGGAATCTGCCTTTTTCCGAAATCATACGGCCCCA 50

1 -------------------------------------------------- 42
2 AGGTCCAAACGGTGAAGGTACCTTAGCTCGCATCCGCATAGTTTTCGCTA 100
3 AGGTCCAAACGGTGAAGGTACCTTAGCTCGCATCCGCATAGTTTTCGCTA 100
4 AGGTCCAAACGGTGAAGGTACCTTAGCTCGCATCCGCATAGTTTTCGCTA 100
5 AGGTCCAAACGGTGAAGGTACCTTAGCTCGCATCCGCATAGTTTTCGCTA 100
6 AGGTCCAAACGGTGAAGGTACCTTAGCTCGCATCCGCATAGTTTTCGCTA 100

1 -------------------AATACAGTTTTTTCGCGCAGTCCAGCCAGTC 73
2 TAGTTTTCATCGTTCGCCGCATACAGTTTTTTCGCGCAGTCCAGCCAGTC 150
3 TAGTTTTCATCGTTCGCCGCATACAGTTTTTTCGCGCAGTCCAGCCAGTC 150
4 TAGTTTTCATCGTTCGCCGCATACAGTTTTTTCGCGCAGTCCAGCCAGTC 150
5 TAGTTTTCATCGTTCGCCGCATACAGTTTTTTCGCGCAGTCCAGCCAGTC 150
6 TAGTTTTCATCGTTCGCCGCATACAGTTTTTTCGCGCAGTCCAGCCAGTC 150
```

Figure S5. Sequence alignment of the C-terminus of *pfkA*. Line 1 was the wild-type. Lines 2-6 were putative insertion mutants identified by colony PCR. The sequence confirmed insertion of the ssrA tag.

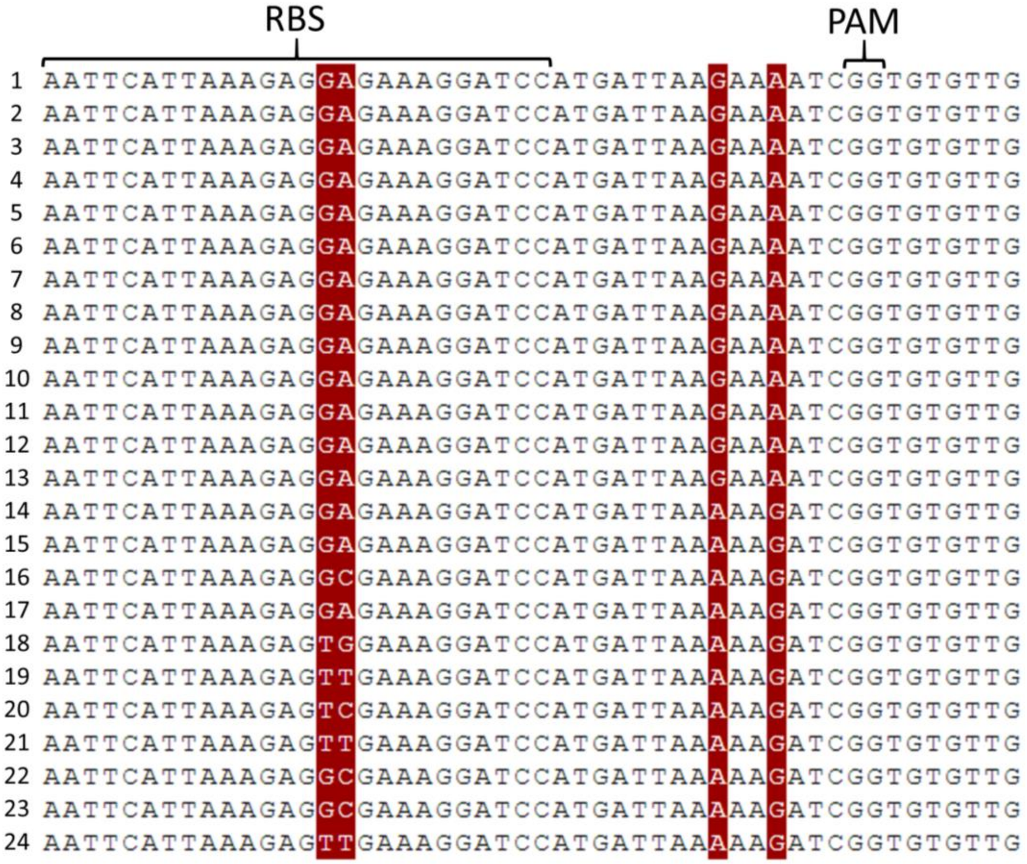

Figure S6. Sequence alignment of 23 putative *pfkE* RBS mutants. Line 1 is the wild-type sequence. Lines 2-13 were putative mutants that had the wild-type sequence. Lines 14,15, and 17 had the silent mutations but possessed the wild-type RBS sequence. Lines 16, 18-24 possessed the silent mutations and mutations in the RBS.

Table S1. Plasmids constructed in this study.

Plasmid Name	Origin of Replication	Antibiotic Resistance	Plasmid Description
pKDsg-pfkAEnd	pSC101	Spec/Strep	Protospacer targeting downstream of *pfkA*
pKDsg-Ack	pSC101	Spec/Strep	Protospacer targeting *ack*
pKDsg-Apt	pSC101	Spec/Strep	Protospacer targeting *apt*
pKDsg-rpoB	pSC101	Spec/Strep	Protospacer targeting *rpoB*
pKDsg-p15A	pSC101	Spec/Strep	Protospacer targeting the p15A origin of replication
pCas9	p15A	Cm	Nuclease positive *cas9*
pCas9cr1	p15A	Cm	pCas9 with ssrA tag
pCas9cr2	p15A	Cm	pCas9cr1 with constitutive *tetR*
pCas9cr4	p15A	Cm	pCas9cr2 with 10-fold reduced RBS

Table S2. Primers used for construction of sgRNA plasmids

Primer Name	Oligonucleotide Sequence
Pkdseq1	ccaattgtccatattgcatca
betaR	tttataacctccttagagctcga
P15AF sgRNA	ctatcgtcttgagtccaaccgttttagagctagaaatagcaag
P15A ptet	ggttggactcaagacgataggtgctcagtatctctatcactGA
pfka end F	GTATtaaTGATTTCGGAAAAgttttagagctagaaatagcaag
pfka end Rptet	TTTTCCGAAATCAttaATACgtgctcagtatctctatcactGA
aptR ptet	GCTTActggAAGACCCGAAAgtgctcagtatctctatcactGA
aptF sgRNA	TTTCGGGTCTTccagTAAGCgttttagagctagaaatagcaagtta
ackF sgRNA	AATAAACAGGAAGCGGCTTTgttttagagctagaaatagcaag
ackR ptet	AAAGCCGCTTCCTGTTTATTgtgctcagtatctctatcactGA

Table S3. Primers used for genotyping PCR

Primer Name	Oligonucleotide sequence
aptFmut	gcgatgtcaccagcttagtct
aptR	cgtccaccaccagaactttg
ackFmut	GAAAATGGACGGCAATAAAGACT
ackR	ACATAGAAGTGGCTGGTGCC
ack Up F	CATGCGCTACGCTCTATGG
ack Down R	CGCCTTTGCGTTCCATTGC
pfkA Down F	cgctatcgatctgctgct
pfkA Down R	gcagaattcatgtaggcctg

Table S4. Oligonucleotides used for chromosomal mutations

Primer Name	Oligonucleotide sequence (*indicates phosphorothioate bond)
rpoB	C*C*AGCAAGTCGATGCTGAGAGCGTAAGCTTTCGGGTCTTAGACTAAGCTGGTGACATCGCG GAAAAGAATGCCG*G*G
ackmut2	C*C*CGAAGCACGTATCAAATGGAAAATGGACGGCAATAAAGACTAAGCGGCTTTAGGTGCA GGCGCCGCTCACAG*C*G
ackCD	G*T*TAGTACTGGTTCTGAACTGCGGTAGTTCTTCACTGGTTATCCCAACCAACGAAGAACTGG TTATCGC*G*C
pfkaRBS	C*G*CATCACCGCCGCTTGTCAACACACCGATCTTTTTAATCATGGATCCTTTCNNCTCTTTAATG AATTGGCTAAACAA*C*T
